# Attentional Selection and Allocation to Alarm Signals in Complex Environments: The New Electrophysiological Evidence

**DOI:** 10.3390/brainsci16010012

**Published:** 2025-12-22

**Authors:** Jia Zhang, Yang Yang, Bingkun Li

**Affiliations:** 1 School of Psychological and Cognitive Sciences, Beijing Language and Culture University, Beijing 100083, China; zhangjia@blcu.edu.cn (J.Z.);; 2 Key Laboratory of Language and Cognitive Science (Ministry of Education), Beijing Language and Culture University, Beijing 100083, China; 3 National Key Laboratory of Human Factors Engineering, China Astronaut Research and Training Center, Beijing 100094, China

**Keywords:** EEG/ERP, alarm, attentional selection/allocation, N2pc, SPCN

## Abstract

**Background/Objectives:** This study explore attentional selection and allocation during alarm signal processing in complex environments. **Methods:** Adopting the dual-task paradigm combining Visual Alarm Task and Main Task with EEG recording, a total of 120 participants were recruited into two experiments with the different presentation order of two tasks. **Results:** Results showed that lateral targets in the Visual Alarm Task induced significant N2 posterior contralateral (N2pc, 200–250 ms) and sustained posterior contralateral negativity (SPCN, 250–500/450 ms) in the parieto-occipital region. N2pc correlated with alarm detection speed and Search Score (the individual search ability), while SPCN correlated with the Subjective Workload Analysis Technique (SWAT) Score. When the Main Task preceded the Visual Alarm Task, the Main Task load modulated attentional selection and allocation—the load of the Main Task moderated the effect of subsequent alarm-elicited sustained negativity on the SWAT Score. **Conclusions:** These findings revealed the functional separation of attentional processes in the cognitive control of alarm signals in complex environments. The study provides new electrophysiological evidence for multi-task attentional allocation and implications for alarm design in high-risk systems.

## 1. Introduction

In high-risk or high-load operational contexts (e.g., driving, flying, nuclear power plant operations, or industrial monitoring), operators often need to perform multiple tasks simultaneously. Specifically, they must focus on the continuous operation of the ongoing task while simultaneously monitoring anomalous information (e.g., alarms) that could appear at any time [1,2,3]. This concurrent multi-task context requires individuals to dynamically allocate attention with limited cognitive resources. As a result, when alarm signals appear, individuals cannot re-allocate attention effectively, thereby giving rise to significant safety risks [1,4,5]. Therefore, understanding how attention resources are allocated to anomalous information (e.g., alarms) in the complex environment holds important theoretical and practical significance.

Research in cognitive psychology and neuroscience has revealed that in dual-task scenarios, individuals must allocate attentional resources across tasks or alternate between them [6,7]. The resulting performance decline, known as the dual-task cost, reflects the limitations of these shared cognitive resources. Specifically, when individuals execute two tasks with high cognitive demands at the same time, the accuracy and speed of their behavioral responses were both notably impaired [8,9]. Furthermore, electroencephalography (EEG) and event-related potential (ERP) technologies offer a tool with high temporal resolution to investigate such attentional allocation. Previous research indicated that mutual interference appears more likely when two tasks share a neurobiological basis. In a dual-task study, participants needed to respond precisely to frequently presented visual stimuli (Task 1) and simultaneously extract information from long-term memory (Task 2). It was found that Task 2 elicited interference in neural electrical activity within the gamma band (>40 Hz), and the brain regions affected by the interference included areas in the Task 1 network, particularly the multiple demand network [10].

In the field of the attentional process by alarm, numerous studies have demonstrated that a number of typical ERP components are capable of sensitively reflecting individual behavioral performance. Early ERP components such as the mismatch negativity (MMN) reflect the early perceptual detection of deviant stimuli [11,12]. On the other hand, P300 is considered a crucial index to assess attention distribution and working memory updating [13]. Under single-task conditions, attention-related electrophysiological indicators (e.g., P300) during the alarm signal disposal stage can effectively predict the behavioral performance of the alarm task. In addition, an increase of P300 amplitude typically implies that more attentional resources are assigned to target stimuli, and it is significantly associated with higher detection accuracy and quicker response speed [1,14]. Notably, previous studies using the paradigm of presenting targets laterally found that N2-posterior-contralateral (N2pc) and the subsequent sustained posterior contralateral negativity (SPCN) specifically characterize attentional retrieval and further attentional processing [15,16], compared with ERP indicators (such as P300) that reflect generalized attention levels. The N2pc typically occurs roughly 200 milliseconds (ms) after stimulus onset, with its amplitude and latency serving as reliable indicators of the spatial attentional orientation [17,18]. In visual search and target detection tasks, an earlier N2pc latency and a larger amplitude often imply quicker and more precise target localization [19,20]. SPCN appears immediately after N2pc, reflecting the sustained attentional maintenance by some special targets, and it might correlate closely with visual working memory load [15,21]. The effective disposal of alarm signals relies on not only the rapid attentional detection within an extremely short time, but also critical decision-making. Thus, N2pc and SPCN may be more suitable for characterizing the individual attentional selection and allocation to alarm signals in the high-cognitive-load specific environments with the random presentation of non-central visual field alarm signals.

Furthermore, when individuals perform the complex task (the main task) simultaneously, the attentional selection and allocation differ significantly from that under the single-task condition. Behavioral studies have shown that when attentional resources are fully occupied by the ongoing task (the main task) in complex environments, it becomes difficult for new stimuli (e.g., unpredictable alarm signals) to obtain sufficient attentional selection and allocation. As a result, alarm signals might fail to be detected effectively or handled correctly, resulting in *Inattentional Blindness/Deafness* [9,22]. However, how attentional resources are allocated under such circumstances remains insufficiently understood. Electrophysiological studies have shown that when the task load increases, the amplitude of the P300 induced by target stimuli usually decreases, and its latency extends [1,2,5]. These studies demonstrate that attentional distribution is limited, and stimulus processing is postponed or weakened. In other words, the workload level of the main task is a key factor influencing attentional allocation [2,5,23,24]. However, it remains unclear how attentional allocation is modulated by the workload level. Thus, investigating how the perceptual workload level regulates the allocation of attentional resources in the alarm signal disposal phase contributes to understanding the neural mechanism of multi-task interference.

It is of note that previous studies on alarm tasks in multi-task scenarios focused exclusively on situations in which the main task and the alarm task occur in a specific sequence, without considering the situation where the processing of an alarm signal is temporarily deferred. Therefore, in the simulation of real operational scenarios with the suspended alarm signal, it remains unknown whether the attention-related electrophysiological indicators by alarm could predict the behavioral performance. Meanwhile, it needs further investigation as to how the main task workload could regulate the attentional allocation to the suspended alarm task. Accordingly, this study focused on examining whether and how the attentional allocation by alarm is affected by the concurrent tasks in the simulated complex environment.

Specifically, using the dual-task paradigm designed to simulate real operational scenarios, we systematically examined the behavioral and electrophysiological characteristics of alarm signal disposal with/without individuals performing a concurrent main task. Based on the hypothesis of limited attention resources and the cognitive control-based load theory [25], we formulated two core hypotheses. First, under single-task conditions—when alarm signal disposal is not disrupted by a concurrent main task—the EEG indices associated with attentional selection (i.e., N2pc and SPCN) would reliably predict performance on the alarm task. Second, when the execution of the main task leads to postponing of alarm signal disposal, attentional resources would be largely occupied, making it difficult for a single alarm-related EEG indicator to independently predict behavioral outcomes. Instead, the attentional allocation pattern would be modulated by the main task workload. Alarm-related electrophysiological indices can effectively predict behavioral performance when the main task workload is low, reflecting a reallocation of attentional resources toward alarm signal disposal. Our study not only advances the theoretical understanding of human multi-task processing and attentional allocation but also provides new neurobiological evidence to inform alarm-system design and operator performance prediction in high-risk operational environments.

## 2. Experiment 1

To investigate the impact of the simultaneous occurrence of alarm signals and the complex scenario task within a close time interval on cognitive attention processes, we presented the simulated visual alarm signal in the peripheral visual field (Visual Alarm Task), while presenting the simulated complex scenario task in the central visual field (Main Task).

### 2.1. Materials and Methods

#### 2.1.1. Participants

In Experiment 1, a total of 60 participants were recruited according to the following standards: (a) normal or corrected-to-normal vision; (b) no history of head injury resulting in loss of consciousness; (c) no organic diseases, neurological disorders, or other severe health issues; (d) right-handedness; (e) no consumption of psychotropic drugs or substances within 24 h prior to the experiment. Eleven participants were not included in subsequent analyses (one participant had a task accuracy below 50%, two participants had less than 40 trials under any condition due to excessive noise in EEG signals, e.g., frequent eye movements or head motions, and eight participants failed to take the accompanying cognitive tests or dropped out of the experiment halfway). Eventually, 49 participants were included in the analysis (23 males, 26 females, aged 18–35 years old). All participants were recruited via the Beijing Language and Culture University, and the experiment obtained approval from the Ethics Committee of the university. Written informed consent was obtained from all participants according to the Declaration of Helsinki.

#### 2.1.2. Experimental Procedure

##### EEG Task

In Experiment 1, which was based on the dual-task paradigm, the Visual Alarm Task (in the peripheral visual field) and the Main Task (in the central visual field) were presented asynchronously. Participants were required to respond only to the Visual Alarm Task, which was presented first, while EEG were recorded. The task was presented with the PsychToolbox software package (http://psychtoolbox.org, accessed on 1 May 2024) running on the MATLAB (R2023b) platform and displayed on a DELL P1917S monitor. The brightness of all colors utilized in the experiment was assessed with the Spectroradiometer JETI spectraval 1501 (JETI Technische Instrumente GmbH, Jena, Germany). Specific RGB parameters had been adjusted to ensure the luminance consistency of these colors.

The Visual Alarm Task (presented in the peripheral visual field) consisted of the Alarm Reminder Period (simulating the state where the alarm signal has not appeared or the participant has not responded) and the Alarm Search Task (simulating the disposal of the alarm signal). In the Alarm Search Task, ten circulars (3° visual angle of each stimulus, where the visual angle of the circular width was 0.5°) with randomly oriented upper or lower openings (1° visual angle) were presented (including 9 Landolt and 1 rectangle with an opening). These ten visual stimuli were randomly arranged in a circle centered on the screen (all stimuli were 12° visual angle away from the screen center and presented for 100 ms). One of the Landolt was red (serving as an irrelevant salient distractor, with specific colors from green, blue, red, yellow), while the other 9 stimuli were gray (RGB: 188, 188, 188). Participants were required to judge the direction of the rectangle’s opening: they pressed the up-arrow key on the keyboard (if the gray rectangle’s opening faced upward) or the down arrow key (if the gray rectangle’s opening faced downward) using the index or middle finger of their right hand. The Alarm Reminder Period also served as a randomized SOA (stimulus onset asynchrony) between the Alarm Search Task and the Main Task. During the Alarm Reminder Period, gray circular rings (RGB: 188, 188, 188) continuously flickered at 2 Hz or 4 Hz in the peripheral visual field. The visual angle and distribution position of these circular rings were consistent with those of the ten stimuli in the visual Alarm Search Task. In Experiment 1, the duration of the Alarm Reminder Period was randomly set between 800 ms and 900 ms.

**Figure 1:**
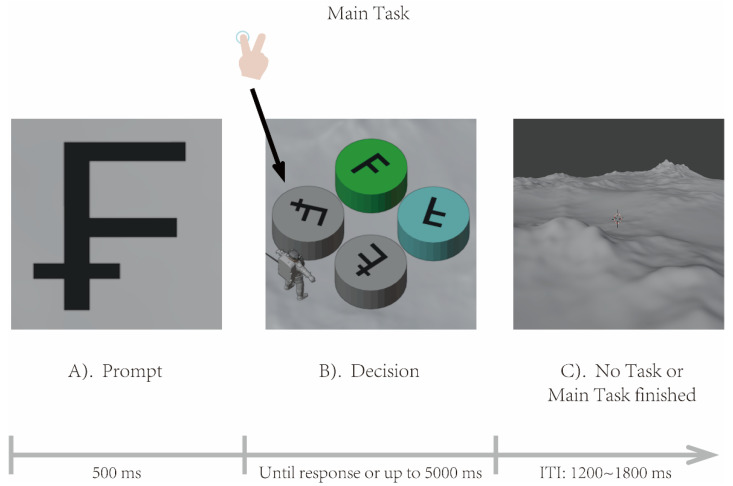
The procedure of the Main Task. In the central visual field, the procedure of the Main Task is as follows: (**A**) A target character cue was first displayed (lasting 500 ms). (**B**) A simulated person with a specific direction and four cylinders with specific colors and symbols was presented in a virtual 3D scene. If the target cylinder was on the left of the person with the specific direction, participants pressed the left arrow key on the keyboard; otherwise, they pressed the right arrow key. The scene was displayed for a maximum of 5000 ms until response. (**C**) After the judgment finished, a no-task scene stimulus was shown (for a random period of 300–800 ms).

For the Main Task (complex scenario task) in the central visual field, a target character cue was first displayed (cue lasting 500 ms with 1.5° visual angle), and then a simulated person with a specific direction and four cylinders with specific colors and symbols was presented in a virtual 3D scene (1.5° visual angle). If participants were required to respond to the Main Task, they needed to find the location of the cylinder marked with the target character based on the specific viewpoint of the simulated person in the virtual 3D scene (participants were not required to respond to the Main Task in Experiment 1). If the target cylinder was on the left of the simulated person facing the specific direction, they pressed the left arrow key on the keyboard using the index finger of their right hand; otherwise, they pressed the right arrow key using the middle finger of their right hand. The scene was displayed for a maximum of 5000 ms until response. After the judgment finished, a non-task scene was shown (for a random period of 300–800 ms). Figure 1 illustrates the procedure of the Main Task. During the cue phase, only one character was randomly displayed at the screen center to indicate the target for the subsequent judgment phase. The virtual 3D scene of the judgment phase was as follows: (a) The four cylinders were placed fixed at symmetrical positions (top, bottom, left, right) centered on the visual field (0.5° visual angle away from the screen center). Two of the cylinders were randomly chosen to be yellow (RGB: 234, 234, 21; 176.9 cd/m^2^), green (RGB: 32, 223, 32; 129.6 cd/m^2^), blue (RGB: 138, 244, 244; 177.5 cd/m^2^), or red (RGB: 234, 21, 21; 137.5 cd/m^2^), while the other two cylinders were gray (RGB: 188, 188, 188; 146.3 cd/m^2^). (b) Four characters were randomly allocated to the top of the four cylinders at different rotation angles (four characters in high-load condition—the prompted character and its mirror, the similar character and its mirror, and four characters in low-load condition—the prompted character and its mirror, the distinct character and its mirror. Each stimulus had a visual angle of 0.5°). (c) The simulated person (with a stimulus visual angle of 0.5°) always faced the screen center and appeared randomly between two adjacent cylinders (lower left, lower right, upper left, upper right), approximately 1° visual angle away from the screen center. In addition, the background of the Main Task exhibited only grayscale variations, with an average luminance of 146.3 cd/m^2^. All images used in the Main Task were generated by the Blender software (Version 4.3.1.0, https://www.blender.org/, accessed on 1 May 2024).

**Figure 2:**
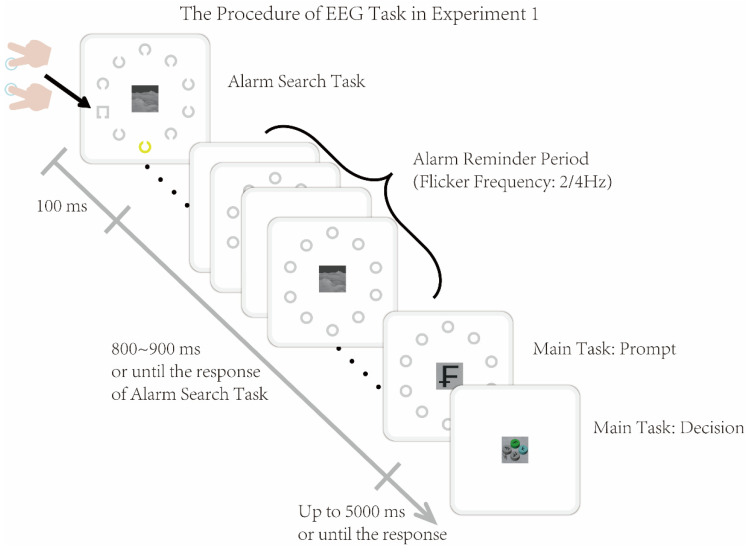
The dual-task procedure in Experiment 1. In each trial, the Alarm Search Task was presented first for 100 ms, followed by an Alarm Reminder Period lasting 800–900 ms. The Main Task started to be presented during the last 500 ms of the Alarm Reminder Period, and the maximum total duration of a single trial was 6000 ms.

Experiment 1 consisted of 4 blocks, with a total of 322 trials, where the block order was balanced between participants. In each trial, the Alarm Search Task was presented first for 100 ms, followed by an Alarm Reminder Period lasting 800–900 ms. The Main Task started to be presented during the last 500 ms of the Alarm Reminder Period, and the maximum total duration of a single trial was 6000 ms (illustrated in Figure 2). In the Alarm Search Task, the target box appeared in the left visual field, right visual field, and midline position for 112, 100, and 112 trials, respectively. The target and the salient color distractor were presented randomly, and their consecutive presentation in the same visual field or at the same position did not exceed 3 times. In the Alarm Reminder Period, ten stimuli flickered at either 2 Hz or 4 Hz randomly, with 162 trials for each flicker frequency. In the Main Task, half of the trials involved the cylinder with the target character being gray (relevant condition: consistent with the color of the target rectangle in the Alarm Search Task), and the other half involved the cylinder being of other colors (irrelevant condition: inconsistent with the color of the target rectangle in the Alarm Search Task). Additionally, all trials were grouped into high load (50%) and low load (50%) by the Main Task. The content, color, and position of the character on the target cylinder were all presented randomly, with the number of repeated presentations not exceeding 2 times.

Participants were seated in a dimly illuminated, soundproof, and electrically shielded laboratory, maintaining a distance of around 70 cm from the computer screen. In the experiment, participants were instructed to keep their gaze fixed on the Main Task in the center of the screen. Once the Alarm Search Task appeared, they were allowed to make key-press responses during the display of the Alarm Reminder Period and the Main Task, with no requirement to respond to the Main Task. Subsequently, they proceeded to the next trial, with an inter-trial interval (ITI) of 300–800 ms. Through practice, participants could quickly respond to the Alarm Search Task. The actual total duration of Experiment 1 was roughly 20 min, and a short break of around 2 min was arranged between blocks.

##### Cognition Tests

Participants were required to complete a Subjective Workload Analysis Technique (SWAT) questionnaire, which assesses the time load, mental effort load, and psychological stress load [26]. The SWAT Score was used as an index of subjective workload during the EEG task. After at least 24 h, they took the Symbol Search (SS) test. During the test, participants were required to identify feature-matched symbols in the search set within the given time limit. In the subtest of the Wechsler Adult Intelligence Scale (WAIS-IV, Chinese version, which is a standardized and structured intelligence assessment), the score of the Symbol Search test is known to be quite suitable for reflecting the target search ability of adults [27].

#### 2.1.3. Electroencephalography Recording and Preprocessing

EEG signals were recorded using a SynAmps2 8050 amplifier (NEURO SCAN, Inc., El Paso, TX, USA) with 64-channel EEG cap (standard 10–20 system). Among the electrodes, VEOG and HEOG electrodes used a bipolar reference, while the other electrodes used the REF electrode online reference. The impedance of all channels was kept below 5 kΩ during the EEG recording, with a sample rate of 1000 Hz. Offline preprocessing was performed with a self-developed MATLAB script that incorporated a function of the EEGLAB toolbox [28].

The data were re-sampled to 250 Hz and subjected to 0.1–30 Hz band-pass filtering using the FIR filter. After interpolating bad electrodes, all electrodes except VEOG and HEOG were re-referenced to the average signal of bilateral mastoid electrodes (M1/2). Trials containing artifacts and incorrect responses were excluded through visual inspection of the EEG data. Independent Component Analysis (ICA) was performed on all electrodes except VEOG and HEOG using the function of runica in EEGLAB, while the number of ICA components was reduced to 20 by combining Principal Component Analysis (PCA) to more accurately identify ocular artifacts. The continuous data were segmented into epochs ranging from –200 to 600 ms, with baseline correction applied using the −200 to 0 ms time window.

To further eliminate ocular artifacts, the following steps were taken: (1) Identifying eye-related components through ICA and removing the ICA components associated with ocular artifacts. (2) Excluding trials where the potential of HEOG electrodes exceeded ±50 µV or the potential of VEOG electrodes exceeded ±75 µV during the entire epoch. Meanwhile, trials were excluded where the potential of any electrode exceeded 100 µV. If the number of valid trials for a participant under any condition was less than 40, the participant was also excluded.

#### 2.1.4. Data Analysis

In the EEG task, the reaction time (RT) and accuracy (ACC) of correct response trials in the Alarm Search Task were analyzed. For EEG data, based on previous studies, the current study selected the attention-specific indicator N2pc to focus on the alarm-specific attention process. Starting from the onset of the Alarm Search Task, independent sample *t*-tests were conducted on a continuous 50 ms window until the end of the epoch to examine the significance of adjacent channel clusters, and the false discovery rate (FDR) correction was applied to the significant results. N2pc occurs in the parieto-occipital region during the late phase of the N2 time window locked to the onset of the target stimulus. Based on the visual field of lateralized target presentation, the electrodes of left and right hemispheres were re-divided into target-contralateral and target-ipsilateral hemispheres in trials, and the difference in waveform is obtained by the contralateral-minus-ipsilateral method [17,18]. Pearson correlation analysis was employed to investigate the correlations among EEG indicators, behavioral performance, and cognitive test outcomes, with the confounding variables of gender and age controlled. The statistical significance level was set to *p* < 0.05 (FDR corrected).

### 2.2. Results

#### 2.2.1. EEG Results

**Figure 3:**
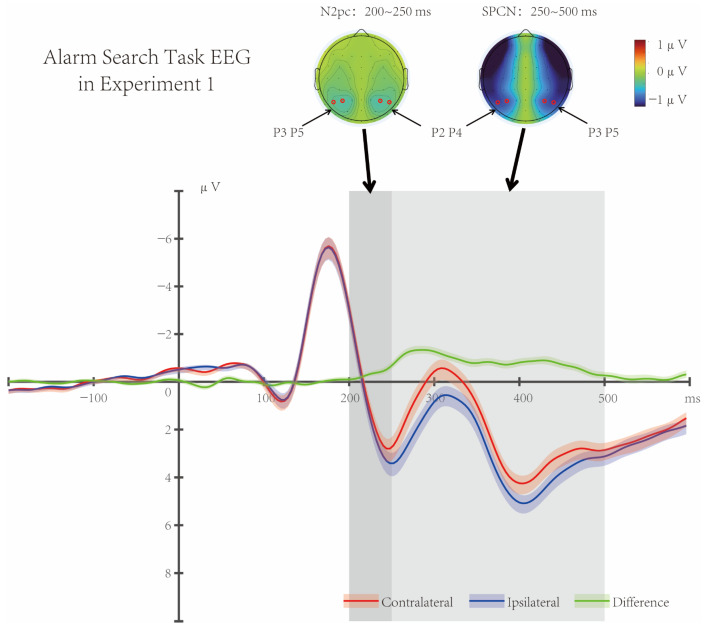
Alarm Search Task EEG in Experiment 1. The target elicited significant negative waves in the parieto-occipital region (electrodes P2/3 and P4/5) from 200 ms to 500 ms (*p*s < 0.003, Cohen’s *d* = 1.023). There was a typical topographic map of early attention shift (N2pc, 200–250 ms) during the N2 time window, followed by a topographic map of sustained attention processing (SPCN, 250–500 ms). Shaded areas indicate the standard error of the mean (SEM).

Results of *t*-tests revealed significant negative waves in the parieto-occipital region (at electrodes P2/3 and P4/5) from 200 ms to 500 ms (*p*s < 0.003, Cohen’s *d* = 1.023). This indicated that the target in the Visual Alarm Task induced attention shift (Figure 3), and the target elicited a typical topographic map of early attention shift (N2pc, 200–250 ms) during the N2 time window, as well as a topographic map of sustained attention processing (SPCN, 250–500 ms).

#### 2.2.2. Correlation of EEG and Behavioral Performance

**Figure 4:**
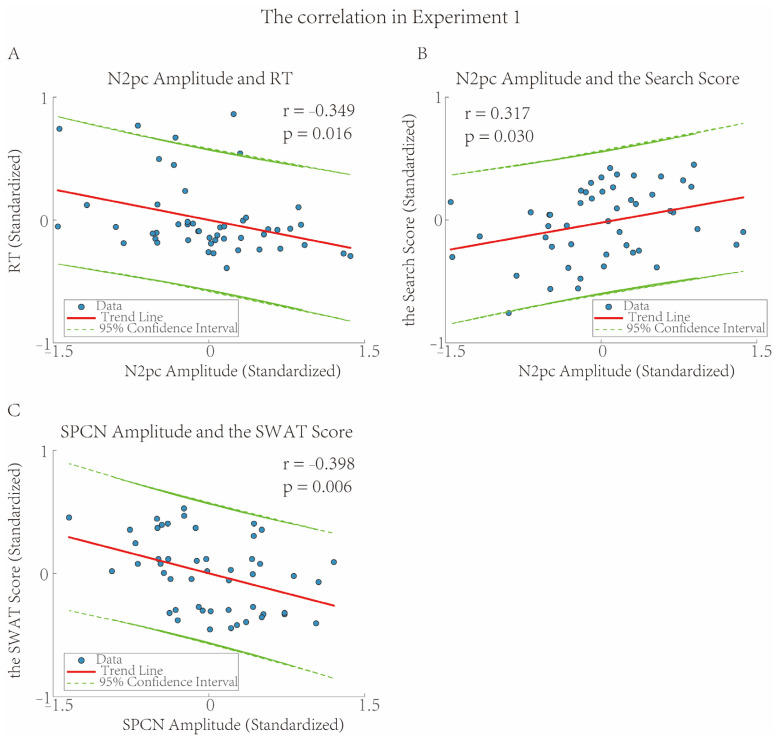
The Correlation in Experiment 1. In (**A**), as the amplitude of N2pc increased, the reaction time became faster (*r* = −0.349, *p* = 0.016, *R*^2^ = 0.122). In (**B**), as the amplitude of N2pc increased, the Search Score was higher (*r* = 0.317, *p* = 0.030, *R*^2^ = 0.101). In (**C**), as the amplitude of SPCN increased, the SWAT Score was lower (*r* = −0.398, *p* = 0.006, *R*^2^ = 0.158).

To investigate how the EEG indicators of the current task could predict behavioral performance, Pearson correlation analysis was used to examine the relationships between two EEG indicators (N2pc, SPCN) and behavioral performance (RT, ACC), respectively. The results showed that as the amplitude of N2pc increased, the RT became faster (*r* = −0.349, *p* = 0.016, *R*^2^ = 0.122, FDR corrected, Figure 4A). There were no significant correlations between other EEG indicators and behavioral performance (*p*s > 0.072, FDR corrected).

#### 2.2.3. Correlation of EEG and Cognition Tests

To investigate the cognitive abilities reflected by the EEG indicators of the current task, Pearson correlation analysis was employed to examine the relationships between the two EEG indicators (N2pc, SPCN) and the cognitive tests (Search Score, SWAT Score). The results showed that as the amplitude of N2pc increased, the Search Score was higher (*r* = 0.317, *p* = 0.030, *R*^2^ = 0.101, FDR corrected, Figure 4B). In addition, as the amplitude of SPCN increased, the SWAT Score was lower (*r* = −0.398, *p* = 0.006, *R*^2^ = 0.158, FDR corrected, Figure 4C). There were no significant correlations between other EEG indicators and cognitive test indicators (*p*s > 0.080, FDR corrected).

### 2.3. Discussion

In real-world scenarios, once the alarm is handled, the continuous alarm reminder ceases. In the current study, after participants finished the Alarm Search Task, the alarm reminder disappeared; subsequently, participants’ cognitive processes during the Main Task did not actually involve any alarm-related attentional processes. Based on this, the current experiment only focused on the attentional processes of the Alarm Search Task.

According to previous studies, depending on the differences in post-attentional cognitive processes after specific tasks, the target-induced attentional shift (N2pc) was usually followed by further attentional processing or entry into the working memory stage [15,21]. In Experiment 1, the Visual Alarm Task appeared prior to the Main Task, and the Alarm Reminder Period occurred in the SOA phase between the Alarm Search Task and the onset of the Main Task, which required further attentional processing. Under the simulated alarm signal mode of the “previous alarm task + subsequent continuous flashing alarm reminder”, the EEG results showed that the parieto-occipital negative waveform induced by lateral targets in the window of 200–250 ms (N2pc) represented the selective attention, while the subsequently sustained posterior contralateral negative (SPCN) represented the process of further attentional processing.

The results of the correlation analysis can further illustrate that the two-stage negative waveform induced by lateral targets in the parieto-occipital region serve different cognitive functions during the alarm signal disposal and alarm reminder processes. Specifically, the RT, which represents target search speed, and the Search Score, which reflects the individual attentional ability, were only correlated with the N2pc induced by lateral targets. This was consistent with previous research findings [19,20], which indicated that N2pc characterized the search for alarm signals in the current task.

The correlation between the SWAT Score and the EEG indicator (SPCN) reflects the process of cognitive resource allocation and investment. The SWAT Score represents the individual workload in the current task. Whether an alarm signal can undergo further attentional processing is usually related to the remaining cognitive resources. Specifically, the lower the cognitive load, the more cognitive resources available for further attentional processing, which is reflected in the larger amplitude of the SPCN for alarm signals in EEG recordings.

## 3. Experiment 2

Experiment 1 focused on the scenario where the Visual Alarm Task preceded the Main Task. After participants completed the Alarm Search Task, the alarm reminder disappeared, and the subsequent Main Task was not affected by the alarm signal. To further investigate the influence of the attentional process of the Visual Alarm Task on that of the Main Task, Experiment 2 focused on the scenario where the Main Task preceded the Visual Alarm Task. In this case, the Main Task was presented together with an alarm reminder. After completing the Main Task, participants were required to resume and finish the previously pending Visual Alarm Task.

### 3.1. Materials and Methods

#### 3.1.1. Participants

To avoid any influence from Experiment 1 on the current study, a further 60 participants were now recruited using the same inclusion criteria as in Experiment 1. Eleven participants were excluded from the analysis of Experiment 2 (one participant had less than 40 trials under any condition due to excessive noise in their EEG signals, such as frequent eye movements or head movements, and 10 participants failed to participate in the cognitive tests). Eventually, a total of 49 participants were included in the analysis (23 males, 26 females, aged 19–29 years). All participants were recruited through the Beijing Language and Culture University. Written informed consent was obtained from all participants according to the Declaration of Helsinki.

#### 3.1.2. Experimental Procedure

**Figure 5:**
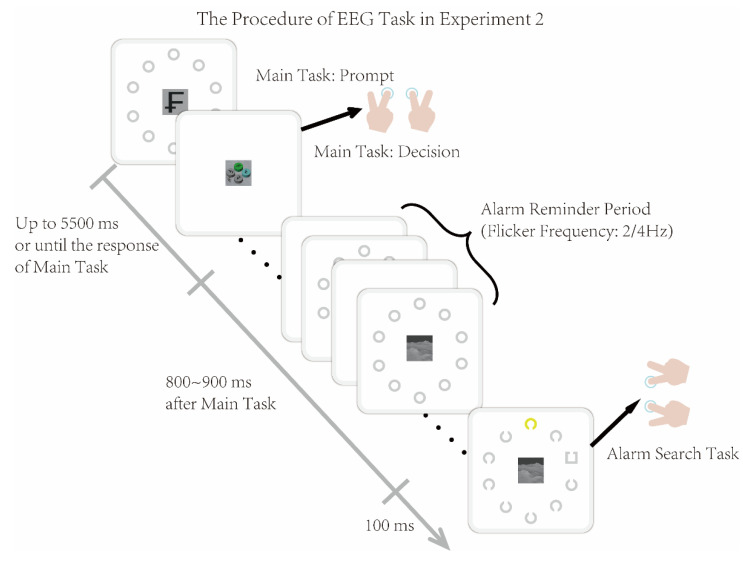
The procedure of Experiment 2. The Main Task and the Visual Alarm Task (the Visual Alarm Task included the sequential presentation of the Alarm Reminder Period and the Alarm Search Task) were sequentially presented in each trial. The parameters and process of each sub-task were consistent with those in Experiment 1.

Based on the dual-task paradigm, Experiment 2 presented the Main Task and the Visual Alarm Task sequentially (the Visual Alarm Task included the sequential presentation of the Alarm Reminder Period and the Alarm Search Task), as illustrated in Figure 5. The parameters and process of each sub-task were consistent with those in Experiment 1.

To further explore the relevant effects, Experiment 2 was divided into two conditions—target-relevant and target-irrelevant—by setting whether the color of the cylinder containing the target in the Main Task matched the color of the target box (gray) in the Alarm Search Task. A randomized SOA was set between the Main Task and the Alarm Search Task, with this interval serving as the Alarm Reminder Period. During this period, ten gray circulars in the peripheral visual field were presented and continuously flickered, followed by the Alarm Search Task.

Experiment 2 also consisted of 4 blocks, with a total of 322 trials. In each trial, a target character cue was first presented (500 ms), followed by the Main Task (presented for a maximum of 5000 ms until the response to the Main Task). After the Main Task disappeared, the non-task scenario was presented in the central visual field and remained until the end of the trial. At the same time, following the disappearance of the Main Task, the Alarm Search Task appeared in the peripheral visual field after a random interval of 800–900 ms and was displayed for 100 ms. The current trial ended after the response to the Alarm Search Task, while the inter-trial interval (ITI) ranged from 300 to 800 ms.

Participants were instructed to respond to the Main Task and Alarm Search Task sequentially. After practice, the total duration of the entire experiment was approximately 40 min, with a short break of about 2 min arranged between blocks.

#### 3.1.3. Electroencephalography Recording and Preprocessing

This was consistent with Experiment 1.

#### 3.1.4. Data Analysis

This EEG task analyzed the RT and ACC of correct response trials based on Experiment 1; the current study examined the lateral targets elicited N2pc in the Alarm Search Task. Starting from the onset of the Alarm Search Task, independent sample *t*-tests were conducted on a continuous 50 ms window until the end of the epoch to examine the significance of adjacent channel clusters, and the FDR correction was applied to the significance results.

Pearson correlation analysis was used to explore the correlations between EEG indicators, behavioral performance, and cognitive tests. Furthermore, a trial-wise correlation analysis method was adopted to investigate the effects of the Main Task and Alarm Reminder Period on the subsequent Alarm Search Task. Specifically, for each participant, Pearson correlation was used to compute trial-by-trial correlation coefficients between the conditions (Main Task—cognitive load, target relevance; Alarm Reminder Period—flicker period, flicker frequency) and the behavioral performance (ACC and RT) of the Alarm Search Task within the same trial. ANOVA was then performed on the correlation coefficients of all participants, and the significance of the *p*-value indicated the consistency of the correlation coefficient [29]. The FDR correction was applied to the significance results of the correlation analyses.

Finally, by comparing the correlation results of Experiment 1, a moderation model was used to explore how the Main Task modulates the relationship between ERP indicators and the individual workload in the Alarm Search Task. The confounding variables (age and gender) were controlled in both correlation analyses and moderation analyses. The statistical significance level was set at *p* < 0.05 (FDR corrected).

### 3.2. Results

#### 3.2.1. EEG Results

**Figure 6:**
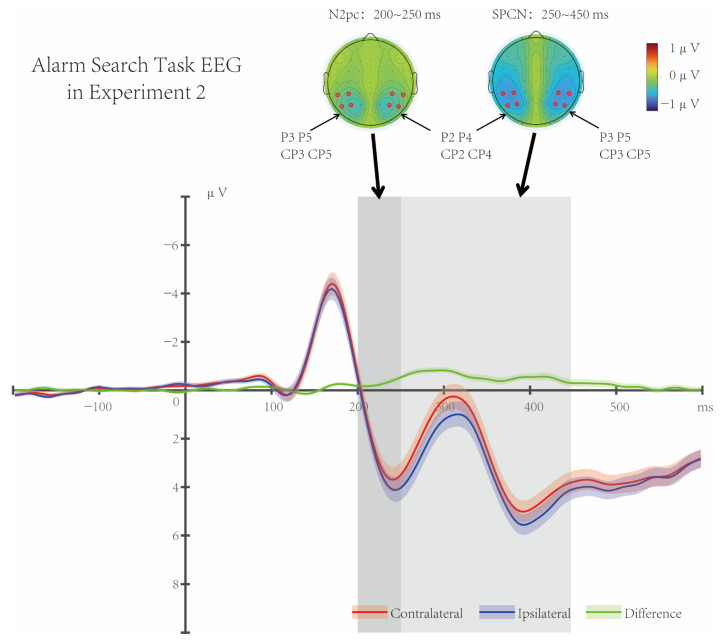
Alarm Search Task EEG in Experiment 2. The target elicited significant negative waves in the parieto-occipital region (electrodes P2/3, P4/5, CP2/3, CP4/5) from 200 ms to 450 ms (*p*s < 0.003, Cohen’s *d* = 0.839). Similar to Experiment 1, there was a typical topographic map of early attention shift (N2pc, 200–250 ms) during the N2 time window, followed by a topographic map of sustained attention processing (SPCN, 250–450 ms). Shaded areas indicate the standard error of the mean (SEM).

Similar to Experiment 1, *t*-tests showed that lateral targets in the Alarm Search Task induced significant and sustained negative waves in the parieto-occipital region (electrodes P2/3, P4/5, CP2/3, CP4/5) (200–450 ms, *p*s < 0.003, Cohen’s *d* = 0.839, Figure 6). During the N2 time window, the targets elicited a typical topographic map of early attentional shift (N2pc, 200–250 ms), as well as a topographic map of sustained attentional processing (SPCN, 250–450 ms).

#### 3.2.2. Subject-Wised Correlation of EEG and Behavioral Performance

**Figure 7:**
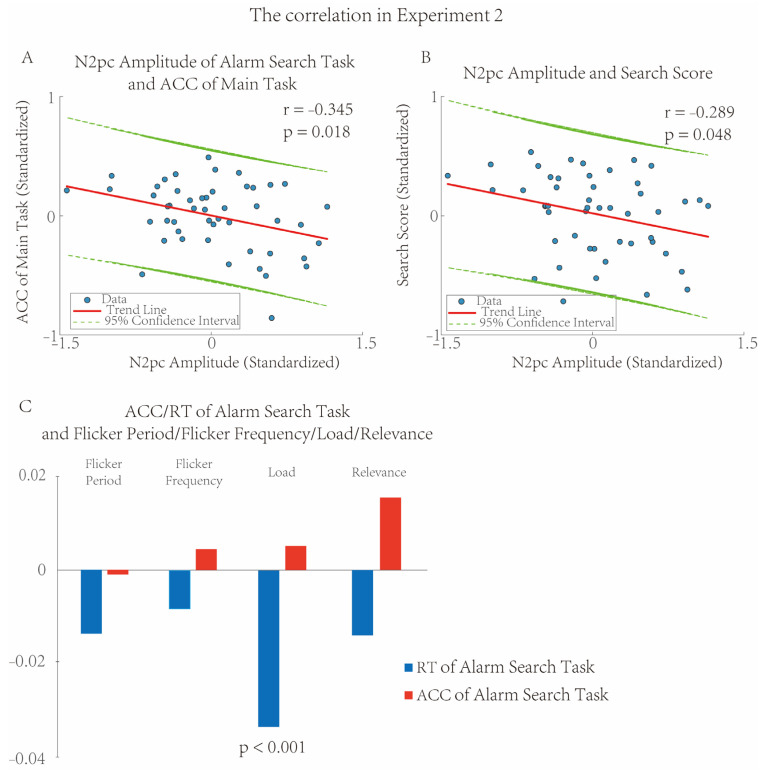
The Correlation in Experiment 2. Subject-wise Correlation of EEG and behaviors displayed an increase in N2pc amplitude, accompanied by a higher ACC of the Main Task (*r* = −0.345, *p* = 0.018, *R*^2^ = 0.119, (**A**)) and a higher score in the Search Score (*r* = −0.289, *p* = 0.048, *R*^2^ = 0.084, (**B**)). Trial-wise Correlation showed the RT of the Alarm Search Task was slower with the higher load of the Main Task (F_(1,45)_ = 27.299, *p* < 0.001, *η*^2^*_p_* = 0.378, (**C**)).

To investigate how the EEG indicators of the Alarm Search Task predict behavioral performance and cognitive abilities, Pearson correlation analysis was used to examine the relationships between two EEG indicators (N2pc, SPCN) and other indicators—including behavioral performance (RT, ACC) and cognitive tests (search ability, SWAT Score). The results showed that an increase in N2pc amplitude was accompanied by a higher ACC of the Main Task (*r* = −0.345, *p* = 0.018, *R*^2^ = 0.119, FDR corrected, Figure 7A) and a higher Search Score (*r* = −0.289, *p* = 0.048, *R*^2^ = 0.084, FDR corrected, Figure 7B). After FDR correction, no other significant correlations were observed (*p*s > 0.227).

In addition, supplementary ANOVAs were conducted to examine the effects of load level and task relevance on both behavioral (RT) and electrophysiological measures (sustained negativity), as well as their potential interaction. This helps to elucidate the role of perceptual complexity in attentional selection and allocation. Specifically, load level (high/low) and task relevance (irrelevant/relevant) were entered as independent variables, and behavioral and EEG indices were treated as dependent variables in the 2 × 2 ANOVA. Detailed results are reported in the Supplementary Materials.

#### 3.2.3. Trial-Wise Correlation of Main Task/Alarm Reminder Period and Alarm Search Task

The subject-wise correlation results in the Alarm Search Task differed from that in Experiment 1, and this difference may be attributed to the effects of the previously presented Main Task and Alarm Reminder Period. To further investigate how the conditions of the pre-presented Main Task and the flicker duration of the Alarm Reminder Period modulate the subsequent behavioral performance of the Alarm Search Task, a trial-wise correlation method was adopted. This method focused on the relationship between the preceding experimental conditions (Main Task—cognitive load, target relevance; Alarm Reminder Period—flicker period, flicker frequency) and the behavioral performance (RT, ACC) of the Alarm Search Task within the same trial. The results showed the RT of the Alarm Search Task was slower with the high load of the Main Task (F_(1,45)_ = 27.299, *p* < 0.001, *η*^2^*_p_* = 0.378, Figure 7C). After FDR correction, no other significant correlations were observed (*p*s > 0.098).

#### 3.2.4. The Moderating Effect of Main Task Load

Compared with Experiment 1, no direct correlation was found between N2pc/SPCN and the individual workload (SWAT Score) in Experiment 2. Based on the results of the current correlation analysis, the load level of the Main Task may moderate the predictive effect of the sustained negativity (200–450 ms) on the subjective load.

The results of the one-sample *t*-test showed that compared with the low load level, the ACC of the Main Task was lower under the high load level (*p* < 0.001), indicating that the difference in ACC across different load levels in the Main Task reflects the load effect. Based on the following formula, we obtained the Main Task indicator (∆ACC) that reflects the cognitive load:

$$∆ACC={ACC}_{High\;Load\;of\;Main\;Task}-{ACC}_{Low\;Load\;of\;Main\;Task}$$

Therefore, the moderation effect was examined with sustained negativity as the predictor, the SWAT Score as the dependent variable, and ∆ACC as a moderator. In the specific model testing, all variables were centered and standardized and then incorporated into three models, respectively:

- Model 1 included the independent variable (sustained negativity) and two control variables (i.e., age and gender)
- Model 2 added the moderator variable (∆ACC) based on Model 1
- Model 3 further added the interaction term (sustained negativity × ∆ACC) based on Model 2.

By comparing Model 3 with Model 2, a significant interaction effect of ∆ACC on the relationship between the sustained negativity and the SWAT Score was found (*t* = −2.664, *p* = 0.011). This indicated that the effect of the sustained negativity on the SWAT Score showed significant differences at different levels of ∆ACC. The results of the moderation effect analysis are presented in Table 1.

**Figure 8:**
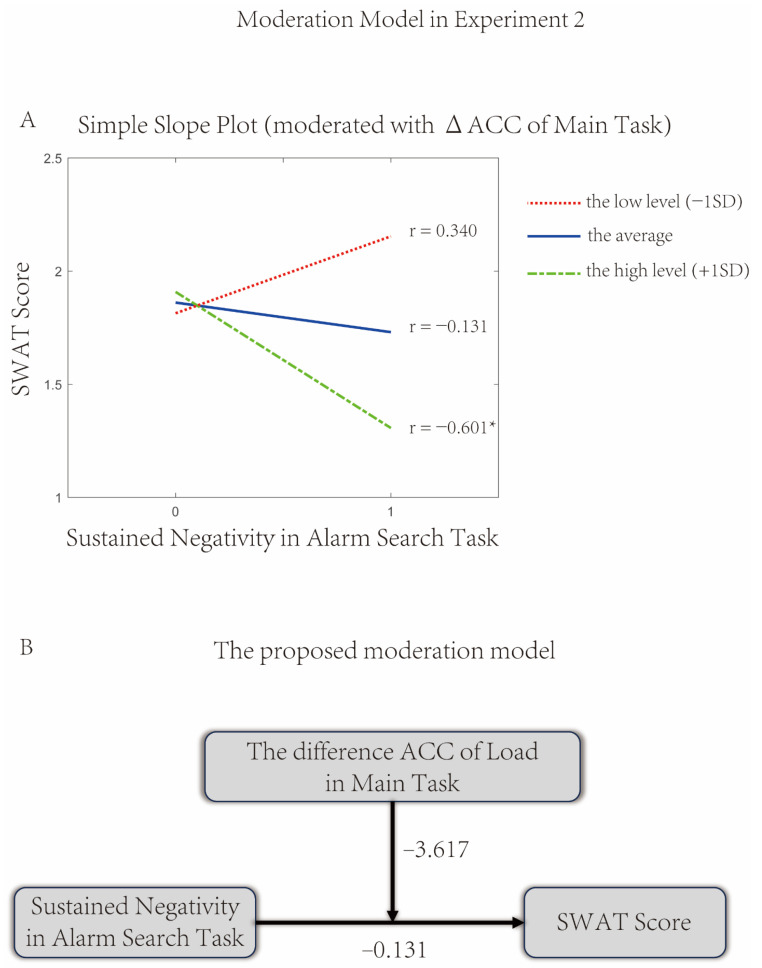
The Moderation Model in Experiment 2. The results of the moderation effect analysis displayed that the effect of the sustained negativity on the SWAT Score showed significant differences in different levels of ∆ACC. In (**A**), it was found participants experienced an effect of smaller sustained negativity on higher SWAT Score (*b* = −3.617, *p* = 0.011) in the higher levels of ∆ACC of the Main Task. The proposed moderation model is specified in (**B**). * *p* < 0.05.

It was found that participants experienced an effect of the smaller sustained negativity on the higher SWAT Score (*b* = −3.617, *p* = 0.011) in the higher levels of ∆ACC of the Main Task. The simple slope plot illustrated the differences in the magnitude (slope) of the effect of the alarm-induced sustained negativity on the individual workload when the moderator variable △ACC was at different levels (Figure 8A). The proposed moderation model is specified in Figure 8B.

Since the higher cognitive load, the lower the ACC of the Main Task, the current moderation model indicated that when the load level of the Main Task was low, a higher sustained negativity was associated with a lower level of subjective cognitive load.

### 3.3. Discussion

In real-world scenarios, if a complex task (Main Task in this study) is being performed, a sudden and persistent alarm reminder will occupy the limited cognitive resources, reducing the success rate of the ongoing task. Meanwhile, the disposal of the alarm signal task is also affected by the cognitive load of the current task, which is reflected in increased subjective cognitive load and the phenomenon of alarm fatigue. In the current study, we focused on the allocation of participants’ attentional resources when the Alarm Search Task occurred after the Main Task. In this case, the Alarm Search Task was influenced by the preceding Main Task and Alarm Reminder Period.

Data analysis showed that in the current experiment, the Main Task appeared before the Visual Alarm Task, and the Alarm Reminder Period occurred during the SOA phase between the Main Task and the Alarm Search Task. Under the current simulated alarm signal mode of the “previous continuous flickering alarm reminder + subsequent alarm task”, lateral targets in the Alarm Search Task elicited the sustained negative waveform in the parieto-occipital region from 200 ms to 450 ms. Based on previous studies on the N2pc/SPCN typical time course and their relationship with cognitive abilities [15,16], Experiment 2 also found that lateral targets induced the significant N2pc (200–250 ms) and SPCN (250–450 ms) in the parieto-occipital region, similar to the EEG results of Experiment 1.

In the results of the subject-wise correlation between EEG indicators and behavioral performance/cognitive abilities in the Alarm Search Task, only a direct association was found between N2pc and both the accuracy of the Main Task and the individual search ability. This indicated that participants’ disposal of alarm signals in the Alarm Search Task was influenced by the preceding Main Task. No direct association was found between SPCN and cognitive abilities, which differs from the results of Experiment 1. Correlation analyses suggested the previously presented Main Task or Alarm Reminder Period further modulated the allocation of attentional resources in the Alarm Search Task.

To describe more precisely the relationship of the previous cognitive processes (the Main Task or Alarm Reminder Period) and the subsequent Alarm Search Task, we adopted a trial-wise correlation approach to investigate the effects of the conditions (Main Task—cognitive load, target relevance; Alarm Reminder Period—flicker period, flicker frequency) on the subsequent behavioral performance of the Alarm Search Task. The results showed the influence of cognitive load in the Main Task, which was mainly reflected in the RT of the Alarm Search Task.

Furthermore, it is necessary to clarify how the cognitive load of the Main Task modulates the relationship between the sustained negativity and the individual workload in the Alarm Search Task. Since the load level is a categorical variable (with only two levels—high load and low load), we first extracted the difference in ACC between high and low load conditions as the load-modulated indicator, i.e., ∆ACC in the Main Task. The moderation model indicated that with limited attentional resources, participants can adjust the allocation of attentional resources between tasks according to the cognitive load, thereby reporting a different subjective perception of workload. For instance, to maintain a relatively low level of subjective cognitive load, when the load level of the Main Task gradually increased, participants re-allocated their attentional resources from the Alarm Search Task to the Main Task.

## 4. General Discussion

To investigate the impact of the simultaneous occurrence of alarm signals and the current complex task within a nearby time window on cognitive attentional processes, we presented the Visual Alarm Task in the peripheral visual field to simulate a visual alarm disposal task, while presenting the Main Task in the central visual field to simulate a complex scenario task. In this study, the two experiments focused on simulated scenarios where the Visual Alarm Task and the Main Task appeared in different sequential orders within a short period. Experiment 1 examined the scenario in which the Visual Alarm Task occurred before the Main Task. Once the alarm signal was detected in the Alarm Search Task, it was handled immediately. After the participant responded, the alarm reminder disappeared, ensuring that the subsequent Main Task was not affected by the alarm signal. We found that attention to the alarm signal directly influenced the performance of the Alarm Search Task. Experiment 2 focused on the scenario where the Main Task preceded the Visual Alarm Task. In this case, the appearance of the Main Task was accompanied by an alarm reminder. After the participant completed the Main Task, they needed to continue completing the pending Visual Alarm Task. We found that when the Visual Alarm Task was completed after the pending stage, attention to the alarm signal was affected by the performance of the previously presented Main Task. Further, we revealed that the selection and allocation of attentional resources between the two tasks was modulated by the load level of the Main Task.

### 4.1. Alarm Signals Detection Requires the Continuous Attentional Allocation in Complex Environments

Inappropriate human-computer interface design increases the cognitive load [30], and recent studies reported inappropriate human–computer interface design of alarm signals. For example, ref. [31] reported that visual alarms have an optimal duration (approximately 200 ms) and an appropriate level of visual contrast. Attentional detection would fail if away from the optimal level of the alarm parameters, reflected in longer reaction times. However, studies relying solely on behavioral data cannot explicitly determine the effect of cognitive load on attentional processes of alarm signals in detail.

ERP has the advantage of high temporal resolution to open the window of the attentional processes. Based on ERP characteristics and their correlation with the individual abilities, we identified multiple attention-related ERP components (N2pc and SPCN) associated with the attentional search for alarm signals and target handling. These components characterized, respectively, the initial capture of attention by the alarm signals and the subsequent further processing process.

This indicated that the successful handling of alarm signals was not a simple single process but rather required the involvement of different types of attentional processes. The alarm signal could be successfully handled associated with a higher-level of the cognitive functional role such as working memory [15]. In Experiment 2, when participants performed a simulated complex scenario task with random alarm signals, the sustained negative waveform induced by the alarm signals was associated with cognitive load. This result suggested the high level of attentional priority alarm signals [32].

Numerous studies have demonstrated that microsaccades can influence lateralized neural activity. For example, previous studies showed that spontaneous microsaccades can induce transient lateralization of EEG alpha activity in accordance with microsaccade direction, exhibiting a spatially oriented pattern [33,34]. If microsaccades are not adequately taken into account, some lateralization activity (e.g., alpha-band oscillation) may be misinterpreted as a purely cognitive effect. In addition, studies focusing on cue-induced eye movements reported modulation of early ERP components, primarily reflected in the amplitudes of the P100 and N100 components [35].

In the present study, although we did not specifically target alpha-band lateralization or early ERP components, we cannot rule out the potential influence of microsaccades. This concern is particularly relevant for Experiment 1. In this experiment, participants were not required to respond to the subsequent Main Task presented at the center of the screen. After target selection in the Alarm Search Task (indexed by the N2pc component, 200–250 ms), participants may not have been strongly motivated to maintain fixation within the central 1.5° visual angle. As a result, increased microsaccades were observed during the subsequent SPCN time window (250–500 ms), introducing a potential confound in the interpretation of the lateralized components observed in Experiment 1. However, microsaccades are unlikely to be the primary factor driving the main conclusions compared with Experiment 2. In Experiment 2, participants were explicitly required to respond to a Main Task presented at the center of the screen within a 1.5° visual angle. No substantial microsaccades were observed during either the early stage (N2pc, 200–250 ms) or the sustained attentional stage (SPCN, 250–450 ms), indicating the robustness of these EEG components and irrelevance of microsaccades.

### 4.2. Alarm Signal Detection Differs from Ordinary Target Search at the Selective Attention Stage

Previous EEG studies on alarm signal failure identified the reduction in the N100 amplitude—the ERP component associated with early sensory processing [36,37]—decreased phase coherence in the alpha band (8–12 Hz) and theta band (4–8 Hz), and the reduction in the P300 amplitude—an EEG indicator linked to the advanced process [1,2,14]. These findings suggested that increased cognitive load contributed to failures in early sensory processing, as well as impairments in subsequent attention/working memory.

However, prior ERP studies showed that the N100 and P300 were associated with general sensory and attentional processing [38,39], and thus may not specifically characterize the phenomenon of alarm signal failure. Earlier laboratory-based studies using the lateralized visual search paradigm found that the N2pc specifically reflected selective attention to targets [17,18], while the latency and amplitude of the N2pc were typically linked to individual attentional capacity and response speed [19,20]. To date, in studies based on a simulated complex scenario, no research has explored how attention to alarm signals affects the performance of alarm signal disposal, nor has any study examined the role and variation patterns of the N2pc and SPCN in tasks involving alarm signal detection and disposal. In the current study, we identified the N2pc elicited by alarm signals. When participants detected and immediately responded to the alarm signals, their attention to the signals directly influenced their performance in handling the alarms. Interestingly, SPCN was still observed without cognitive load. This pattern differs from the findings of ref. [15], which emphasized variations in cognitive load, and it also diverges from results reported in simple target-search paradigms [40]. It indicated that the special significance of alarm signals affected the attention process, compared to ordinary targets.

Perceptual load theory provides a useful framework for further interpreting alarm signal detection in a complex environment [41,42]. According to this theory, perceptual processing proceeds automatically until perceptual capacity is fully occupied. When perceptual load is high, limited perceptual resources are rapidly consumed by the Main Task, thereby restricting further processing of task-relevant stimuli and, to some extent, reducing the inhibition of task-irrelevant information. Within the current dual-task environment, the Main Task involved different levels of perceptual load, while the Alarm Search Task varied in task relevance. For behavioral performance (e.g., RT of Alarm Search Task, seen in the Supplementary Materials), when perceptual load was high, limited cognitive resources were quickly exhausted by the Main task. As a result, the unexpected alarm signal under task-irrelevant conditions could not be effectively suppressed, leading to accelerated response in the Alarm Search Task. In contrast, when perceptual load was low, cognitive resources could be allocated to the current task-irrelevant stimulus, facilitating its suppression and thereby delaying reaction times in the Alarm Search Task. When the alarm signal was task-relevant, attentional resources were not directed toward suppressing the alarm stimulus, and consequently no perceptual load modulation effect was observed. For EEG indices, the alarm stimulus reliably elicited lateralized negativity, indicating that target stimuli could capture spatially selective attention to some extent. However, variations in the load of the Main Task altered participants’ strategies for distributing attentional resources across the two tasks. It modulated the number of attentional resources available for maintaining alarm-related information, as reflected by participants’ subjective workload (SWAT Scores). Specifically, when attentional resources were preferentially allocated to the Main Task, behavioral performance in the Main Task improved, while the level of sustained attention devoted to alarm search targets was constrained. Under conditions in which sufficient attentional resources were required to ensure adequate performance in the attention task, participants reported higher subjective workload. This modulation pathway suggests that the SPCN, as an index of sustained attention, is sensitive to changes in attentional resource allocation across tasks.

From the perceptual load perspective, significant modulation of the early attentional orienting component (N2pc) by cognitive load could not be observed, which might relate to the relatively automatic and robust nature of selective attention. In contrast, later stages of attentional maintenance and processing, as indexed by the SPCN, are more susceptible to variations in load and task demands. This pattern indicates that, in complex multi-task environments, the allocation of attentional resources across different tasks depends not only on the initial selective attention, but more importantly on whether this attention can be sustained, processed, and effectively utilized over time [15]. Taken together, these findings support the view that attentional allocation is a multi-stage, dynamic process, rather than a single, discrete processing stage. This stage-specific dissociation not only provides converging support for perceptual load theory but also carries important practical implications: under high-load conditions; the primary challenge faced by alarm systems may lie not in initial attentional selection, but in securing sufficient sustained attentional resources for effective processing.

### 4.3. The Dual-Task Paradigm Serves as a Tool to Investigate the Attentional Resource Allocation Across Tasks

The dual-task paradigm in this study investigated the attentional allocation across sub-tasks. Thus, the paradigm could be considered as an appropriate tool to simulate human–computer interface scenarios of alarm signals with cognitive conflict. On the one hand, previous studies used the Stroop paradigm [43], the Flanker paradigm [44], and their variants to identify and verify cognitive processing competition and interference during stages such as sensation, attention, decision-making, and execution [45,46]. To some extent, these studies provided evidence for explaining the cognitive processing of alarm signals at various cognitive stages. However, since alarm signals are independent of the currently performing task, the task mechanism of cognitive conflict in real-world scenarios is fundamentally different from the cognitive conflict focused on by the Stroop and Flanker paradigms. Thus, these paradigms cannot be used to examine cognitive conflicts caused by two or more independent tasks in simulated environments. On the other hand, multi-task paradigms, such as the Multi-Attribute Task Battery (MATB), task focusing on the individual abilities of cognitive control and cognitive conflict across multiple cognitive processes. The MATB can be used to explore task difficulty adjustment and multi-task design in complex scenarios [47,48], but they cannot strictly control specific experimental variables.

To clearly distinguish the sources of alarm interference, many studies based on simulated real-task scenarios adopted a dual-task paradigm to focus on factors such as task perceptual load, cognitive load, and alarm signal characteristics [12,49,50]. These studies have revealed the alarm signals’ cognitive mechanisms of perception, attention, memory, and decision-making in complex environments, providing a theoretical basis for optimizing alarm signal design.

In addition, to optimize the effectiveness of dual-task paradigm, alarm signal design must be based on the characteristics of scenarios. For example, auditory alarms are widely used in fields requiring rapid response, such as medical monitoring, because they are unaffected by visual interference and do not require specific attention shifts [50,51]. However, given auditory alarms are omnidirectional and difficult to ignore, the simultaneous occurrence of multiple auditory alarms can increase subjective discomfort [52]. In contrast, the visual channel can simultaneously receive stimuli containing rich spatial information (e.g., object location, shape, and motion trajectory), and visual stimuli (e.g., symbols, colors, text, or moving images) are intuitive and have cross-cultural universality [53]. Under the constraints of visual perceptual load and working memory load, only limited information located in the direction and position of attentional selection can be specifically processed. Therefore, in complex scenarios, the visual channel is well-suited for presenting small amounts of alarm information with specific content [54], such as aircraft instrument warnings, stop signs, or emergency exit indications.

In the current study, a dual-task paradigm was used to simulate the occurrence and response of visual alarm signals at different temporal stages of an ongoing complex task in real-world environments. By simultaneously recording EEG data, the study innovatively identified the mutual influences between multiple tasks, as well as the evidence of the flexible attentional allocation across multiple tasks.

### 4.4. Limitations and Future Directions

There are other limitations in our study. First, the absence of a control condition (e.g., a simple task without alarms) limits the ability to isolate alarm-specific effects. Second, differences in task order between Experiments 1 and 2 may introduce confounds (e.g., cognitive load effects), which could be influenced by practice or sequence bias.

As for future directions, participants were healthy students in our study, which restricts the generalizability of the results to trained professionals such as pilots or air-traffic controllers. Future studies can broaden the participant sample (e.g., experienced operators) to assess whether the findings can be generalized to real-world expertise. Then, the interpretation of SPCN as an index of subjective workload would benefit from comparison with additional physiological indicators of cognitive load (e.g., eye-tracking, pupillometry, heart-rate variability). Future studies can integrate these physiological indicators to strengthen the interpretations of attentional dynamics. Next, although the experimental paradigm was designed to increase ecological validity, it does not fully capture all aspects of real-world operational settings, such as prolonged stress, fatigue, or time pressure. Future work may benefit from integrating these aspects for a more comprehensive investigation. In addition, future work can increase the task variability, including the testing of multiple alarm modalities (auditory, tactile) and varying levels of cognitive load to improve generalizability. Finally, future research could investigate the optimal alarm duration, contrast level, and redundancy strategies as well as other factors of individual differences.

## 5. Conclusions

In this study, simulated dual-task scenarios were introduced to explore the dynamic characteristics of attentional selection and allocation of alarm signals in complex operational environments. Furthermore, the results showed N2pc was primarily associated with alarm detection speed, while SPCN was closely related to subjective workload and was modulated by the load of the concurrent Main Task. These findings demonstrated a functional dissociation between attentional selection and allocation and highlighted the critical role of task load in shaping sustained attentional engagement with alarm signals. This study was considered to deepen the theoretical understanding of multi-task processing and attentional allocation mechanisms, as well as to offer an important neurobiological basis for appropriate human–computer interface design in high-risk alarm operation systems.

## Figures and Tables

**Table 1:** The Result of the Moderating Effect Analysis.

	Model 1	Model 2	Model 3
Constant	2.343 * (2.327)	2.369 * (2.370)	1.861 (1.947)
**Control variables**			
Age	−0.066 (−1.564)	−0.068 (−1.627)	−0.047 (−1.192)
Gender	0.385 * (2.016)	0.398 * (2.101)	0.441 * (2.472)
**Main effect**			
Sustained negativity	−0.131 (−0.868)	−0.128 (−0.850)	−0.131 (−0.928)
∆ACC		0.924 (1.301)	0.362 (0.519)
**Interaction**			
Sustained negativity × ∆ACC			−3.617 * (−2.664)
Sample Size	49	49	49
*R* ^2^	0.162	0.193	0.308
Adjusted *R*^2^	0.106	0.120	0.227
*F* Value	*F*_(3,45)_ = 2.907, *p* = 0.045	*F*_(4,44)_ = 2.637, *p* = 0.047	*F*_(5,43)_ = 3.820, *p* = 0.006
∆*R*^2^	0.162	0.031	0.114
∆*F* Value	*F*_(3,45)_ = 2.907, *p* = 0.045	*F*_(1,44)_ = 1.693, *p* = 0.200	*F*_(1,43)_ = 7.094, *p* = 0.011

Note: Dependent variable is the SWAT Score. * *p* < 0.05 values in parentheses are *t*-values.

## Data Availability

The data supporting this study’s findings are available on request from the corresponding authors, B.L. However, the data are not publicly available because they contain information that could compromise the privacy of the research participants.

## Supplementary Materials

The following supporting information can be downloaded at: https://www.mdpi.com/article/10.3390/brainsci16010012/s1, Figure S1: The interaction between load level and task relevance on RT of the Alarm Search Task.
