# Supplementary material for: Attentional Selection and Allocation to Alarm Signals in Complex Environments: The New Electrophysiological Evidence
Source: Brain Sci. 2025 Dec 22;16(1):12. doi: 10.3390/brainsci16010012 (PMC12838615; doi:10.3390/brainsci16010012)
Supplement: Supplementary file 1 [file brainsci-16-00012-s001.zip › brainsci-4022720-supplementary.pdf]

Supplementary Materials

ANOVAs were conducted in Experiment 2 to examine the effects of load and task relevance on both behavioral (RT) and electrophysiological measures (sustained negativity), as well as their potential interaction. This helps to elucidate the role of perceptual complexity in attentional selection and allocation. Specifically, load (high/low) and task relevance (irrelevant/relevant) were entered as independent variables, and behavioral and EEG indices were treated as dependent variables in a 2 × 2 ANOVA.

First, for reaction time (RT) in the Alarm Search Task, our results showed a significant main effect of load level ($F_{(1, 48)} = 8.988$, $p = 0.004$, $\eta^2_p = 0.158$) as well as a significant load × task relevance interaction ($F_{(1, 48)} = 7.641$, $p = 0.008$, $\eta^2_p = 0.137$). Post hoc analyses showed that the RT of the high load was significantly faster than the low load level in the task-irrelevant condition ($p < 0.001$, Figure S1). These findings were consistent with perceptual load theory (Lavie, 2005; Lavie and Tsal, 1994). Specifically, when perceptual load was high, limited cognitive resources were rapidly consumed by the Main Task, making it more difficult to suppress unexpected alarm signals that occur under conditions unrelated to the task, thereby accelerating the reaction. In contrast, when perceptual load was low, surplus cognitive resources could be allocated to the task-irrelevant alarm stimulus, facilitating its suppression and resulting in slower RT. When the alarm signal was task-relevant, no load-dependent modulation of reaction time was observed. This result showed that attentional resources were not suppressing the task relevant alarm directly.

Second, for sustained negativity in the Alarm Search Task, our results revealed no significant main effects or interaction effects (*ps* > 0.186), indicating that load level or task relevance did not exert a direct influence on sustained attentional maintenance. Instead, these factors might modulate attentional resource allocation through alternative pathways. Given that the effects of load and task relevance were observed only in Experiment 2 (i.e., when participants were required to respond to the Main Task), we did not perform additional repetitive analyses for Experiment 1.

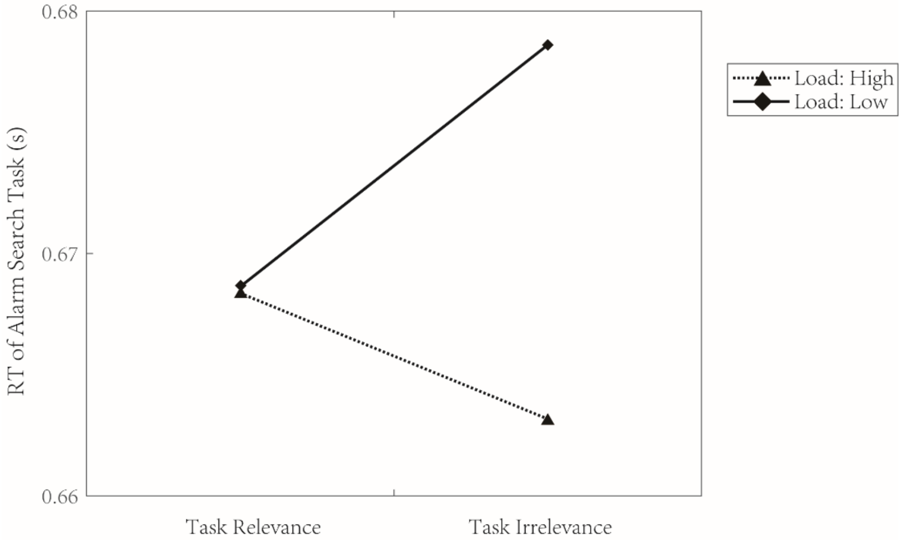

Fig. S1. The interaction between load level and task relevance on RT of the Alarm Search Task. RT of the high load was significantly faster than the low load level in the task-irrelevant condition ($p < 0.001$), while no load-dependent modulation was observed in the condition of task-relevant.

Reference

Lavie, N. (2005). Distracted and confused?: Selective attention under load. *Trends in cognitive sciences, 9*(2), 75-82.

Lavie, N., & Tsal, Y. (1994). Perceptual load as a major determinant of the locus of selection in visual attention. *Perception & psychophysics, 56*(2), 183-197.
